# Knock-Out of *CmNAC-NOR* Affects Melon Climacteric Fruit Ripening

**DOI:** 10.3389/fpls.2022.878037

**Published:** 2022-06-10

**Authors:** Bin Liu, Miguel Santo Domingo, Carlos Mayobre, Ana Montserrat Martín-Hernández, Marta Pujol, Jordi Garcia-Mas

**Affiliations:** ^1^Centre for Research in Agricultural Genomics (CRAG), CSIC-IRTA-UAB-UB, Barcelona, Spain; ^2^Institut de Recerca i Tecnologia Agroalimentàries (IRTA), Barcelona, Spain

**Keywords:** fruit ripening, melon (*Cucumis melo* L.), NAC-NOR, CRISPR, shelf life

## Abstract

Fruit ripening is an important process that affects fruit quality. A QTL in melon, *ETHQV6.3,* involved in climacteric ripening regulation, has been found to be encoded by *CmNAC-NOR*, a homologue of the tomato *NOR* gene. To further investigate *CmNAC-NOR* function, we obtained two CRISPR/Cas9-mediated mutants (*nor-3* and *nor-1*) in the climacteric Védrantais background. *nor-3*, containing a 3-bp deletion altering the NAC domain A, resulted in ~8 days delay in ripening without affecting fruit quality. In contrast, the 1-bp deletion in *nor-1* resulted in a fully disrupted NAC domain, which completely blocked climacteric ripening. The *nor-1* fruits did not produce ethylene, no abscission layer was formed and there was no external color change. Additionally, volatile components were dramatically altered, seeds were not well developed and flesh firmness was also altered. There was a delay in fruit ripening with the *nor-1* allele in heterozygosis of ~20 days. Our results provide new information regarding the function of *CmNAC-NOR* in melon fruit ripening, suggesting that it is a potential target for modulating shelf life in commercial climacteric melon varieties.

## Introduction

Fruit maturation is an important developmental stage because the set of biochemical pathways involved in ripening make the fruit attractive, perfumed, and edible (Bouzayen et al., 2010). In addition, the ripening process also helps seed dispersal (Wang et al., 2020a). There is considerable ongoing research to help understand the complex regulation of this important process (Bouzayen et al., 2010). Based on their ripening behavior, fleshy fruits have been divided into two groups: climacteric and non-climacteric (McMurchie et al., 1972). Climacteric fruits such as tomato are characterized by an ethylene burst accompanied by an increase in respiration at the onset of ripening. In contrast, non-climacteric fruits such as orange lack this ethylene-associated respiratory peak (Paul et al., 2012). Usually, climacteric fruits have a shorter shelf life than non-climacteric fruits (Hiwasa-Tanase and Ezura, 2014), and breeding programs for fruit crops are oriented to increase shelf life to minimize postharvest losses (Payasi and Sanwal, 2010).

Ethylene plays a primary role in initiating climacteric fruit ripening (McMurchie et al., 1972). Its production is low at the pre-climacteric stage, while there is a massive auto-stimulated ethylene production at the onset of the ripening stage. Exogenous ethylene treatment can also induce the ethylene burst at the pre-climacteric stage of climacteric fruits, thereby advancing the ripening process (Hiwasa-Tanase and Ezura, 2014). In contrast, treatment with the ethylene inhibitor 1-methylcyclopropene (1-MCP) delays fruit ripening (Blankenship and Dole, 2003; Watkins, 2006). Antisense-induced repression of ethylene synthesis enzymes in tomato also delays fruit maturation (Hamilton et al., 1990). These results demonstrate the key role of ethylene in regulating ripening in climacteric fruits. Interestingly, some reports have shown that ethylene can also play a role in non-climacteric fruit ripening (Katz et al., 2004; Bouzayen et al., 2010), although in non-climacteric fruit, a low level of ethylene during the whole developmental process is found (Paul et al., 2012). In melon, some fruit ripening processes are independent of ethylene, such as flesh softening, sugar accumulation, or flesh color, that do not change in ethylene-suppressed melon fruit (Flores et al., 2001; Pech et al., 2008), confirming that the control of fruit ripening is a complex trait.

Genes involved in fruit ripening have been largely studied in either climacteric or non-climacteric species (Gapper et al., 2013; Osorio et al., 2013; Lü et al., 2018) and tomato has emerged as a prime model of climacteric fruit ripening (Alexander and Grierson, 2002). Genetic characterization of several ripening-related mutants in tomato has advanced our knowledge of the mechanisms that regulate fruit ripening (Giovannoni, 2007). The *ripening-inhibitor* (*rin*), *non-ripening* (*nor*), and *Colorless non-ripening* (*Cnr*) mutations have been useful to understand the transcriptional regulation of fruit ripening (Vrebalov et al., 2002; Manning et al., 2006; Giovannoni, 2007; Wang et al., 2020b). However, the milder ripening phenotypes observed for the CRISPR/Cas9 knockouts of these three genes have resulted in a re-evaluation of their original proposed role as master regulators of fruit ripening, suggesting that a network of partially redundant components exists to regulate this important biological process (Ito et al., 2017; Gao et al., 2019, 2020; Wang et al., 2019, 2020a). Additional transcription factors involved in the regulation of fruit ripening have also been identified in tomato. The MADS-box transcription factor *TOMATO AGAMOUS-LIKE1* (*TAGL1*; Itkin et al., 2009; Vrebalov et al., 2009) is highly expressed during fruit ripening. *TAGL1*-silenced fruit did not ripe normally, with reduced levels of carotenoids and ethylene. *FRUITFULL* homologues (*TDR4/FUL1* and *MBP7/FUL2*) are also MADS-box transcription factors involved in fruit ripening in an ethylene-independent manner, having redundant functions in cell wall modification (Bemer et al., 2012). *FUL1/2* and *TAGL1* may regulate different subsets of the known RIN targets. *APETALA2a* (*AP2a*) is a negative regulator of tomato fruit ripening, with its silencing causing elevated ethylene production and early fruit ripening (Chung et al., 2010; Karlova et al., 2011). Other NAC proteins have also been found to be involved in regulating ripening, among them *SlNAC1*, *SlNAC3*, *SlNAC4,* and *SlNAM1*, suggesting that a complex regulatory network of fruit ripening exists (reviewed in Liu et al., 2022). For non-climacteric fruit, strawberry is one of the most studied plants (Osorio et al., 2013) and several genes involved in strawberry fruit ripening have recently been identified, including *FaPYR1* (Chai et al., 2011), *FaExp2* (Civello et al., 1999), *FaASR* (Chen et al., 2011), *FaABI1* (Jia et al., 2013), and *FaRIF* (Martín-Pizarro et al., 2021). These studies have provided valuable information on gene function related to regulation of fruit ripening.

Melon (*Cucumis melo* L.) is a suitable model to study fruit ripening, because there are both climacteric and non-climacteric genotypes (Ezura and Owino, 2008). Genetic analysis of a biparental population of the cantaloupe type Védrantais (VED, climacteric) × PI 161375 (SC, non-climacteric) inbred lines indicated that ethylene production and fruit abscission are controlled by two independent loci, *Al-3* and *Al-4* (Périn et al., 2002). In recent studies, a near isogenic line SC3-5-1 derived from the non-climacteric parental lines SC and the inodorus type Piel de Sapo (PS) had a climacteric ripening phenotype, and two QTLs, *ETHQB3.5* and *ETHQV6.3*, were found to be involved in the regulation of climacteric ripening in SC3-5-1 (Eduardo et al., 2005; Moreno et al., 2008; Vegas et al., 2013). Previously, *ETHQV6.3* was found to be encoded by a *NAC* transcription factor *CmNAC-NOR* (MELO3C016540.2), phylogenetically related to the tomato *SlNAC-NOR* (Ríos et al., 2017). TILLING mutants containing non-synonymous mutations in the coding region of *CmNAC-NOR* had a delayed ripening phenotype, suggesting that *CmNAC-NOR* is an important regulator of climacteric ripening in melon. To further investigate the *CmNAC-NOR* function, in this study, we generated and phenotyped CRISPR/Cas9 mutants with different disruption levels.

## Materials and Methods

### Plant Material and Generation of Constructs

The cantaloupe inbred line VED (climacteric) was used in this study. For editing *CmNAC-NOR* in VED, three gRNAs (gRNA1, gRNA2, and gRNA3; Supplementary Table S1) were designed, based on the genomic sequence of *CmNAC-NOR*, using Breaking-Cas (Oliveros et al., 2016). The gRNA1 and gRNA2 sequences were inserted into the vector pBS_KS_Bsa_Bbs_tandem with the *BbsI* and *BsaI* sites, respectively, cut using the *SpeI* and *KpnI* restriction enzymes, and then inserted into the final pB7-CAS9-TPC vector to obtain the gRNA1-gRNA2-CAS9 construct. The same protocol was used to generate the gRNA2-gRNA1-CAS9 and gRNA3-gRNA1-CAS9 constructs. These constructs were transformed into Agrobacterium (AGL-0) and identified by cloning PCR. Cloning vectors were kindly provided by Professor Puchta (KIT, Germany).

### Melon Transformation

Cotyledon transformation was used for melon transformation using Agrobacterium strain AGL-0 as described by Castelblanque et al. (2008) except that the cotyledons were cut as in García-Almodóvar et al. (2017). In brief, half of the proximal parts of the cotyledons from 1-day-old seeds were cut and co-cultured with transformed Agrobacterium for 20 min in the presence of 200 μM acetosyringone. The inoculated explants with Agrobacterium were co-cultured for 3 days at 28°C on regeneration medium supplemented with 0.5 mg/l 6-bencylaminopurine (BA), 0.1 mg/l Indole-3-acetic acid (IAA), and 200 mM acetosyringone. Every 3–4 weeks, the green cluster buds were cut and explants were moved to fresh selection medium in the presence of L-Phosphinothricin (PPT). When the regenerated shoots were tall, they were cut, separated from the explants, and put individually into rooting media in large test tubes. When the rooted plantlets were large enough, a leaf section was cut to identify edited T0 plants.

### Genotyping and Ploidy Test

Genomic DNA was extracted from young leaves of melon plants by an improved CTAB method (Pereira et al., 2018). To genotype the candidate plants, the *CAS9* gene was amplified to confirm that plants were transgenic; then, the target region of the three gRNAs was amplified and sequenced. Primers used in this study are listed in Supplementary Table S1. At the same time, young leaves were harvested and sent to Iribov (Heerhugowaard, Netherlands) for the ploidy test using flow cytometry (FCM).

### Identification of CAS9 Free T_1_ Plants

Diploid T_0_ plants that carried the *CAS9* gene were selected, grown, and self-pollinated to obtain the T_1_ seeds. The T_1_ seeds were germinated and genotyped, and the *CAS9* free plants with or without *CmNAC-NOR* editions were selected for further experiments.

### Fruit Phenotyping

Fruit quality traits, especially those associated to climacteric ripening behavior, were assessed after harvest as previously described (Ríos et al., 2017; Pereira et al., 2018). In brief, the production of ethylene in the fruits was measured from 25 days after pollination (DAP) to when fruit dropped, or 65 DAP when it did not drop, using a non-invasive ethylene quantification method (Pereira et al., 2017). The same method for measuring ethylene (Pereira et al., 2017) was used for ethylene treatment, where 250 ml of 50 ppm ethylene was injected in the bag with the fruit, which was then phenotyped after the bag had been kept closed for 1 week. The production of aroma was detected by olfactory evaluation of fruits from 25 DAP until harvest. The number of days for abscission layer formation were also recorded. External color change during fruit ripening was phenotyped visually. Fruits were weighed at harvest. Soluble solid content was analyzed with a digital hand refractometer (Atago Co. Ltd., Tokyo, Japan). Flesh firmness was measured using a penetrometer (Fruit TestTM, Wagner Instruments).

### Volatiles Analysis

The aroma profiling of the flesh tissue of melon fruits was analyzed with GC–MS as previously described (Mayobre et al., 2021). Briefly, 2 g of frozen flesh was ground, weighed, and added to 20 ml chromatography vials with 1 g of NaCl and 7 ml of saturated NaCl solution containing 15 ppm of 3-hexanone as internal standard. Samples were stored at 4°C a maximum of 7 days. Solid-Phase Micro-Extraction (SPME) was carried out by pre-heating samples for 15 min at 50°C and centrifuging at 250 rpm. The SPME fiber (50/30 μm DVB/CAR/PDMS, Merck^®^, Darmstadt, Germany) was exposed to the vial headspace for 30 min. Splitless injection was used in a 7890A gas chromatograph (GC) equipped with a Sapiens-X5MS capillary column (30 m/0.25 mm/0.25 μm, Teknokroma^®^, Sant Cugat del Vallès, Spain), with 10 min of thermal desorption at 250°C. The oven was set to 50°C for 1 min, then increasing by 5°C/min to 280°C and holding for 5 min. The carrier gas was helium at a head pressure of 13.37 psi. A mass spectrometer (MS) 5975 C (Agilent Technologies^®^, Santa Clara, CA, United States) was coupled to the GC, with a source temperature of 230°C and the quadrupole temperature was set to 150°C. With an untargeted analysis, volatiles were identified by comparison of their mass spectra with the NIST 11 library (NIST/EPA/NIH) and by their Kovats retention index, calculated using a mix of alkanes (C7-C40 in hexane, Merck^®^, Darmstadt, Germany) under the same chromatographic conditions. The relative content of each volatile was estimated by normalizing the peak area to the internal standard peak. A Shapiro–Wilk normality test (*α* = 0.05) and a multiple variable *t*-test were carried out. A Wilcoxon test was used to compare edited plants against wild type.

### Gene Expression Analysis

Total RNA was extracted from flesh tissue at harvest from three biological replicates per genotype (Control, non-edited (NE), *nor-3* and *nor-1*) using the Spectrum Plant Total RNA Kit (Sigma, Burlington, MA, United States). A DNase treatment was performed using Turbo DNase kit (Invitrogen, Waltham, MA, United States). cDNA was synthesized using PrimeScript kit (Takara, Kyoto, Japan). Quantitative RT-PCR was performed in a LightCycler^®^ 480 with LightCycler^®^ 480 SYBR Green I Master (Roche, Basel, Switzerland). Expression data are presented as fold-change (2^-ΔΔCT^; Livak and Schmittgen, 2001), using *CmCYP7* (MELO3C025848.2) as a reference gene for normalization, which was validated in Saladié et al. (2015). Primers used for RT-qPCR are listed in Supplementary Table S1.

### Data Analysis

DNA and protein sequence alignments were obtained with DNAMAN version 7. For the statistical analyses, the R (v3.5.3) software (R Core Team, 2020) was used. ANOVAs and pairwise *t*-test were performed using R package “rstats.” In general, significance was fixed at value of *p* <0.05.

## Results

### Generation of *CmNAC-NOR* Disrupted Mutant Lines by CRISPR/Cas9

**Figure 1:**
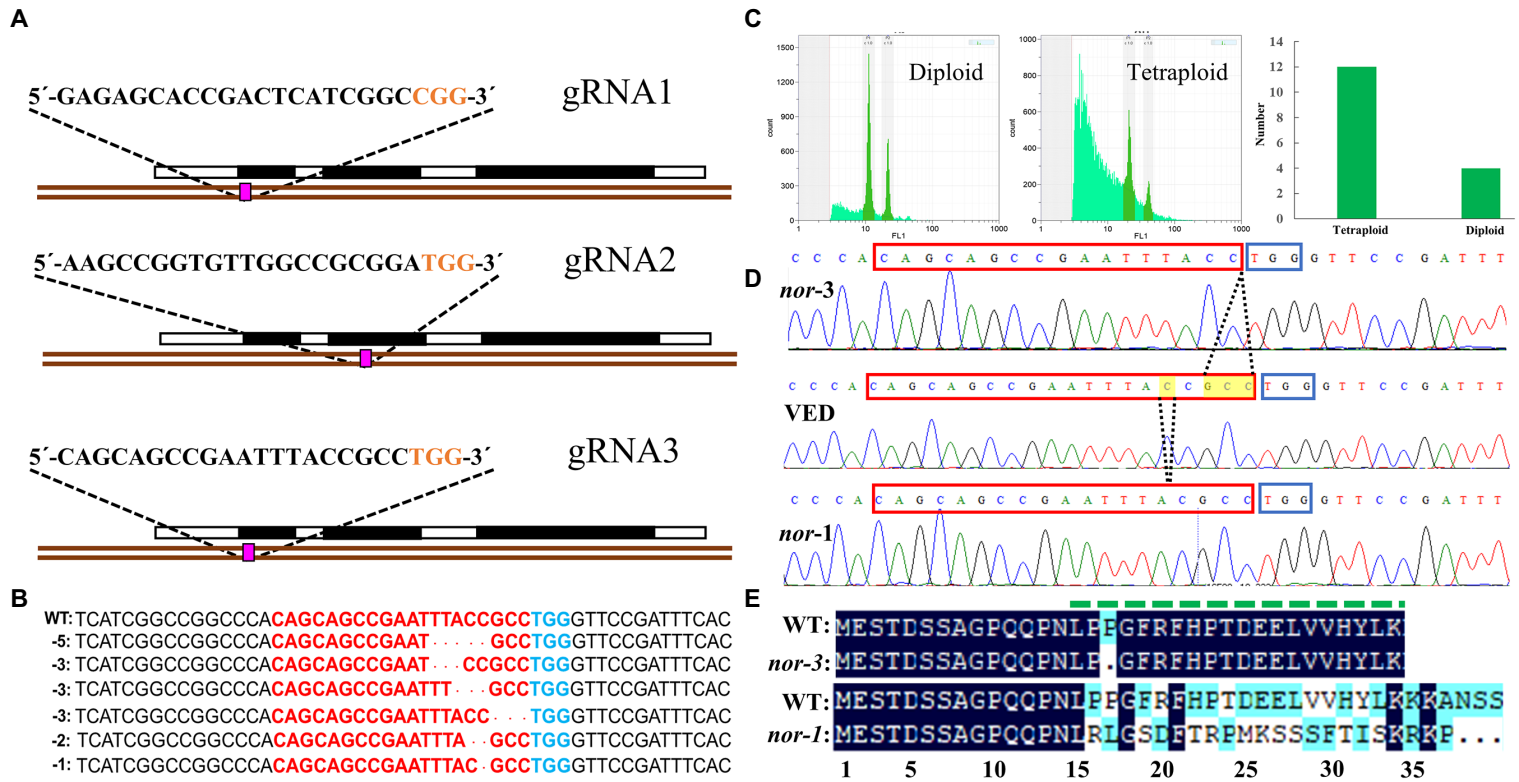
Generation of *CmNAC-NOR* disrupted mutant lines by CRISPR/Cas9. **(A)** Illustration of the *CmNAC-NOR* gene structure and the three gRNAs in exons 1 and 3. **(B)** Six independent mutations at gRNA3 targeting sites. **(C)** Ploidy test of transgenic lines. Left, diploid line NOR-g3-22; Middle, tetraploid line NOR-g3-20; and Right, total number of diploid and tetraploid plants obtained. **(D)** Validated edited lines by Sanger sequencing. The yellow boxes and dotted lines indicate the deleted nucleotides and their position. **(E)** Amino acid alignment of the CmNAC-NOR gRNA3-target region in VED (WT), *nor-3,* and *nor-1*. NAC subdomain A is shown by a dashed green line above the protein sequences.

*CmNAC-NOR* contains three exons and encodes a protein of 353 amino acids (Ríos et al., 2017). In this study, three guide RNAs (gRNAs) that specifically target the first or second exon of *CmNAC-NOR* were designed (Figure 1A). After melon transformation, we obtained 83 T_0_ plants containing the gRNA2-gRNA1-CAS9 and six containing the gRNA1-gRNA2-CAS9 construct, but none of them were edited. In contrast, we obtained 39 T_0_ lines that contained the gRNA3-gRNA1-CAS9 construct, and six mutations at the gRNA3 target site were detected (Figure 1B). As melon tissue culture often induces the generation of tetraploid plants (Ezura et al., 1992), we looked at the ploidy of 15 individuals and found three diploid plants (Figure 1C). These diploid plants contained two different mutations, a-3 bp and a-1 bp deletion, which were named *nor-3* and *nor-1*, respectively (Figure 1D). The −3 bp deletion in *nor-3* results in the loss of the proline in position 17 (Figure 1E), which is predicted as a deleterious change (score:-14.874) by PROVEAN (Protein Variation Effect Analyzer; Choi and Chan, 2015). The −1 bp deletion in *nor-1* results in major changes from amino acid 16 and the generation of a truncated protein of 37 aa (Figure 1E). The functional regions in the NAC subdomain A of the CmNAC-NOR protein were totally disrupted in *nor-1* (Figure 1E), suggesting that *nor-1* is a loss-of-function mutant. In the following studies, we used T_1_ plants of the two lines and VED and T_1_ non-edited (NE) plants as controls.

### Ethylene Production, Volatile Profile, and Gene Expression in *nor* Mutants

**Figure 2:**
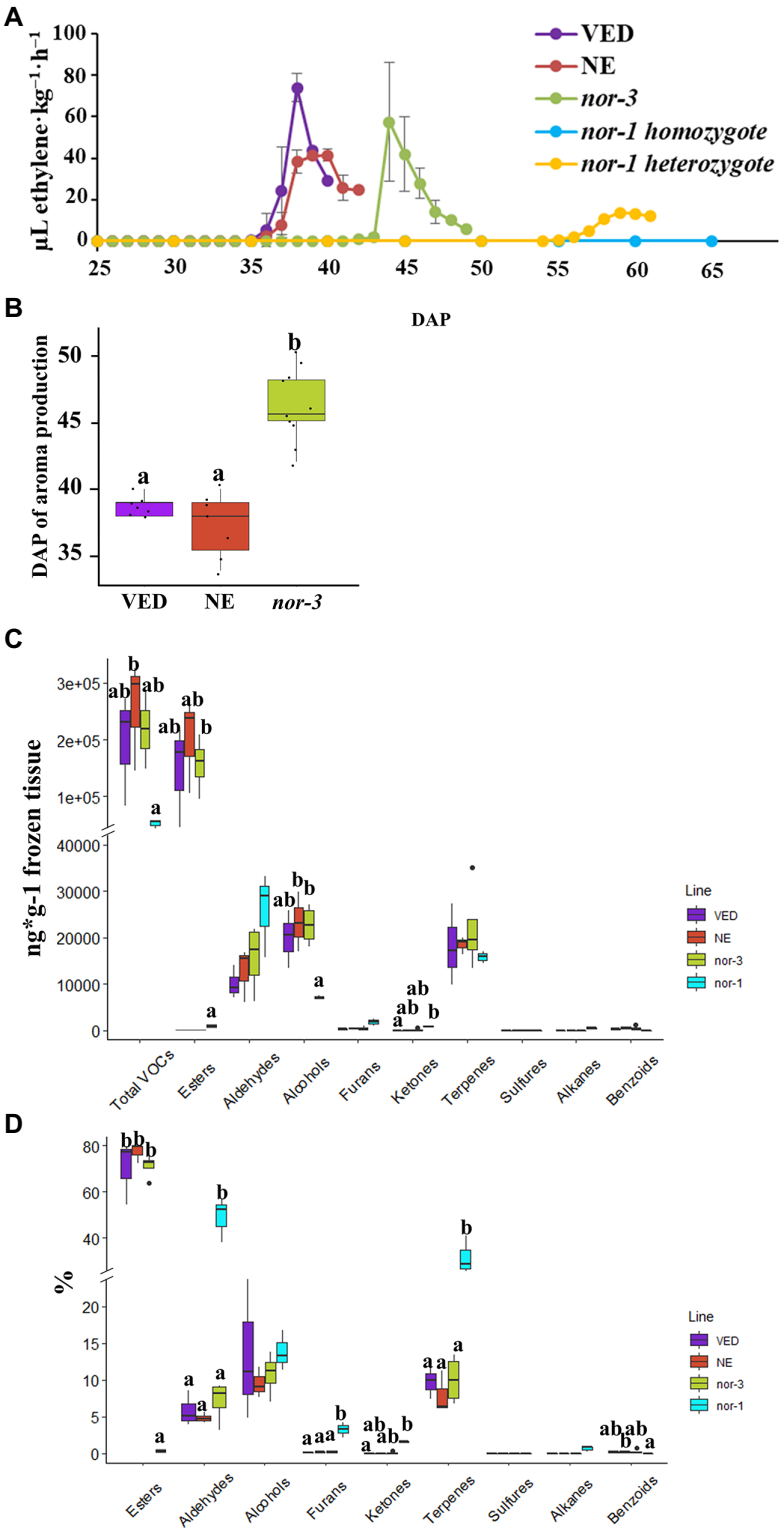
Ethylene production and volatile profile in *nor* mutants. **(A)** Ethylene production in VED, NE (non-edited control), *nor-3*, *nor-1 homozygote*, and *nor-1 heterozygote* according to days after pollination (DAP). Means are plotted ±SD (*n* = 5) except *nor-1* heterozygote (*n* = 1). **(B)** Phenotypic comparison of aroma production in VED, NE, and *nor-3* at harvest. Lower case letters indicate significant differences (*p* < 0.05, *n* > 5). **(C,D)** Relative volatile content in VED, NE, *nor-3*, and *nor-1* at harvest by mass **(C)** and mass percentage **(D)**. Lower case letters indicate significant differences (*p* < 0.05, *n* = 3); large size black dots represent outliers.

Given the key role of ethylene in initiating climacteric fruit ripening (McMurchie et al., 1972), we first compared the ethylene production between the controls and both *nor* mutants. The ethylene production was recorded from 25 DAP, and the results showed a significant delay (~8 days) in the production of ethylene in *nor-3* when compared to VED and NE, but without a significant difference in the amount of ethylene produced (Figure 2A). For the *nor-1* mutant in homozygosis, we did not detect any ethylene production even at 65 DAP (Figure 2A). Interestingly, there was a ~ 20-day delay in ethylene production for the *nor-1* allele in heterozygosis, and the amount of ethylene produced was much lower than with the controls (Figure 2A).

VED is a *cantalupensis* melon, which has intense aroma during climacteric fruit ripening due to the high production of esters (Obando-Ulloa et al., 2008; Mayobre et al., 2021). Therefore, we also compared the aroma production between the controls and both *nor* mutants. In *nor-3* fruits, the aroma production was significantly delayed compared to the controls at the ripe stage (Figure 2B). However, we did not detect aroma production in *nor-1.* To investigate which volatiles were altered in the mutants, we used GC–MS to study the fruit flesh volatile profile. As shown in Figures 2C,D, we found no significant differences in the volatile profile between *nor-3* and controls, but *nor-1* mutants had a completely different profile with a major decrease in total VOCs produced, which explains the lack of aroma by olfactory evaluation. In the *nor-1* mutant, we detected far fewer ester compounds and an increase in aldehydes, furans, and terpenes compared to the wild-type VED (Supplementary Table S2).

To understand the regulation of ripening by *CmNAC-NOR*, we analyzed the expression of genes related to key ripening pathways: ethylene biosynthesis, ester production, flesh softening, and carotenoid production at harvest. Two key genes involved in the biosynthesis of ethylene: 1-aminocyclopropane-1-carboxylate synthase (*CmACS1* MELO3C021182.2) and 1-aminocyclopropane-1-carboxylate oxidase (*CmACO1* MELO3C014437.2) increased their expression during ripening in climacteric genotypes (Saladié et al., 2015). We observed that *CmACS1* and *CmACO1* were expressed in both control lines, VED and NE, at similar levels as the *nor-3* mutant, while their expression was dramatically reduced in the *nor-1* mutant (Supplementary Figure S1). To elucidate the regulation of aroma production by *CmNAC-NOR*, we evaluated two genes involved in ester production, one alcohol dehydrogenase *CmADH2* (MELO3C014897.2; Manríquez et al., 2006) and one alcohol acyl-transferase *CmAAT1* (MELO3C024771.2; El-Sharkawy et al., 2005). The expression of both genes was repressed in the *nor-1* mutant, whereas they were highly expressed in *nor-3* and control lines, which is in agreement with the decrease of esters in the *nor-1* mutant (Supplementary Figure S1; Figures 2B–D). Two polygalacturonases (*CmPGs* MELO3C013129.2 and MELO3C016494.2) involved in melon fruit softening during ripening (Hadfield et al., 1998; Nishiyama et al., 2007; Saladié et al., 2015) were also evaluated and we observed that they were repressed in the *nor-1* mutant compared to *nor-3* and control lines (Supplementary Figure S1). To estimate the effect of *CmNAC-NOR* in carotenoid synthesis, we evaluated the expression of *CmOr* (MELO3C005449.2), a gene involved in beta-carotene accumulation in melon flesh (Tzuri et al., 2015). The results showed that *CmOr* was not differentially expressed when comparing both mutants *nor-1*, *nor-3*, and the control lines VED and NE (Supplementary Figure S1).

### Partially Disrupting *CmNAC-NOR* in *nor-3* Delays Fruit Ripening but Does Not Affect Fruit Quality

**Figure 3:**
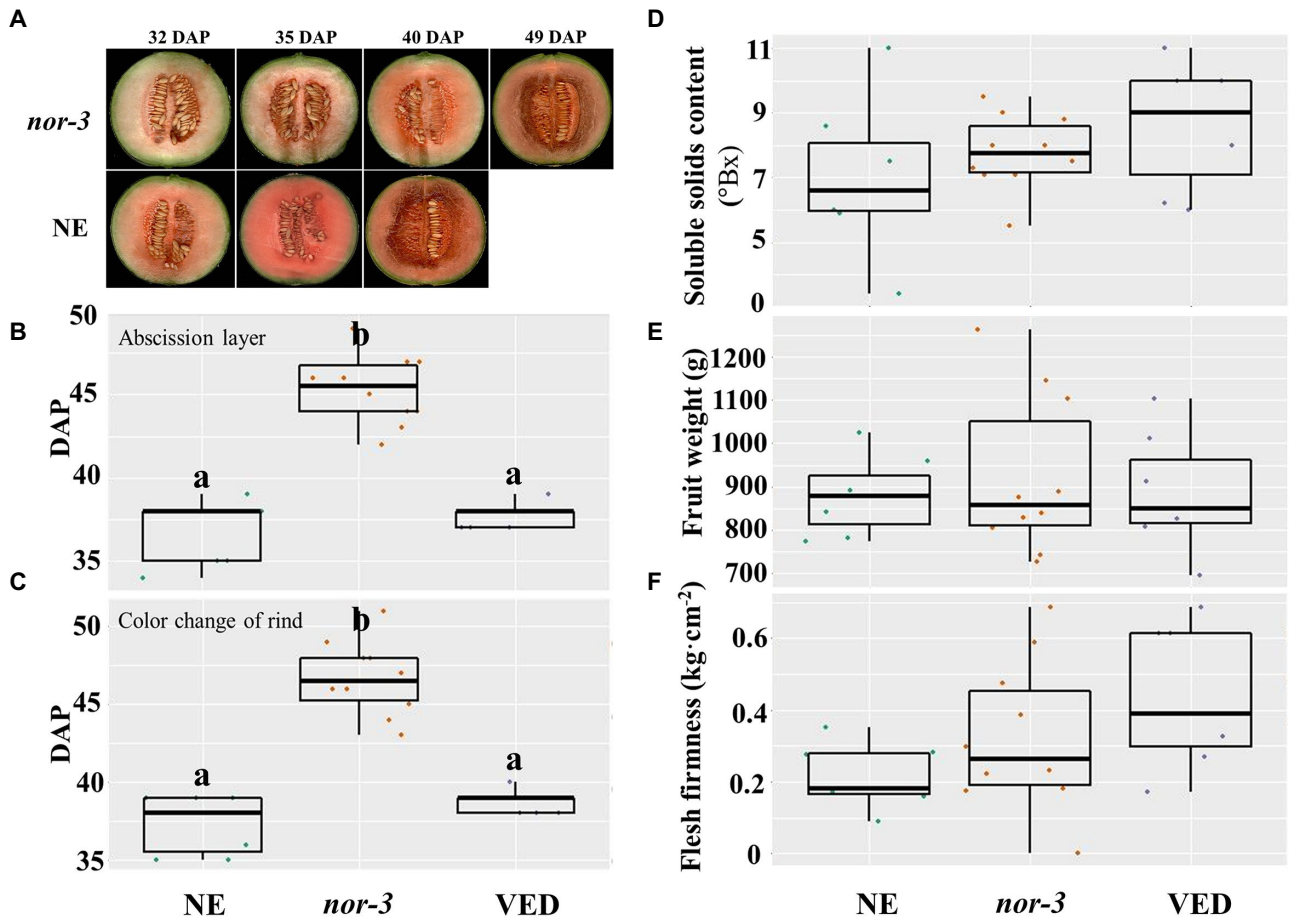
Partially disrupting *CmNAC-NOR* in *nor-3* delays fruit ripening. **(A)** Fruit ripening phenotype of *nor-3* and NE (non-edited control) under natural ripening conditions at days after pollination (DAP). **(B-F)** Phenotypic comparisons according to abscission layer formation **(B)**, color change of the rind **(C)**, soluble solids content **(D)**, fruit weight **(E)**, and flesh firmness **(F)** among NE, *nor-3,* and VED. Lower case letters indicate significant differences (*p* < 0.05, *n* > 5).

The results from ethylene and aroma production suggest that *nor-3* has a delayed ripening phenotype, and similar results were obtained with other ripening-related traits. The flesh color of *nor-3* at 40 and 49 DAP was similar to that of NE at 32 and 40 DAP, respectively (Figure 3A), confirming the 8–9 days ripening delay in *nor-3.* In addition, abscission layer formation (Figure 3B) and rind color change (Figure 3C) of *nor-3* fruit were also significantly delayed compared to the controls at the ripe stage. However, we found no significant difference in the amount of ethylene produced (Figure 2A), which is consistent with our previous findings in *CmNAC-NOR* TILLING mutants (Ríos et al., 2017). We also measured the soluble solids content (SSC, Figure 3D), fruit weight (Figure 3E), and flesh firmness (Figure 3F) at harvest, and no significant differences were detected between NE, *nor-3,* and VED.

### *CmNAC-NOR* Knock-Out in *nor-1* Blocks Climacteric Ripening

**Figure 4:**
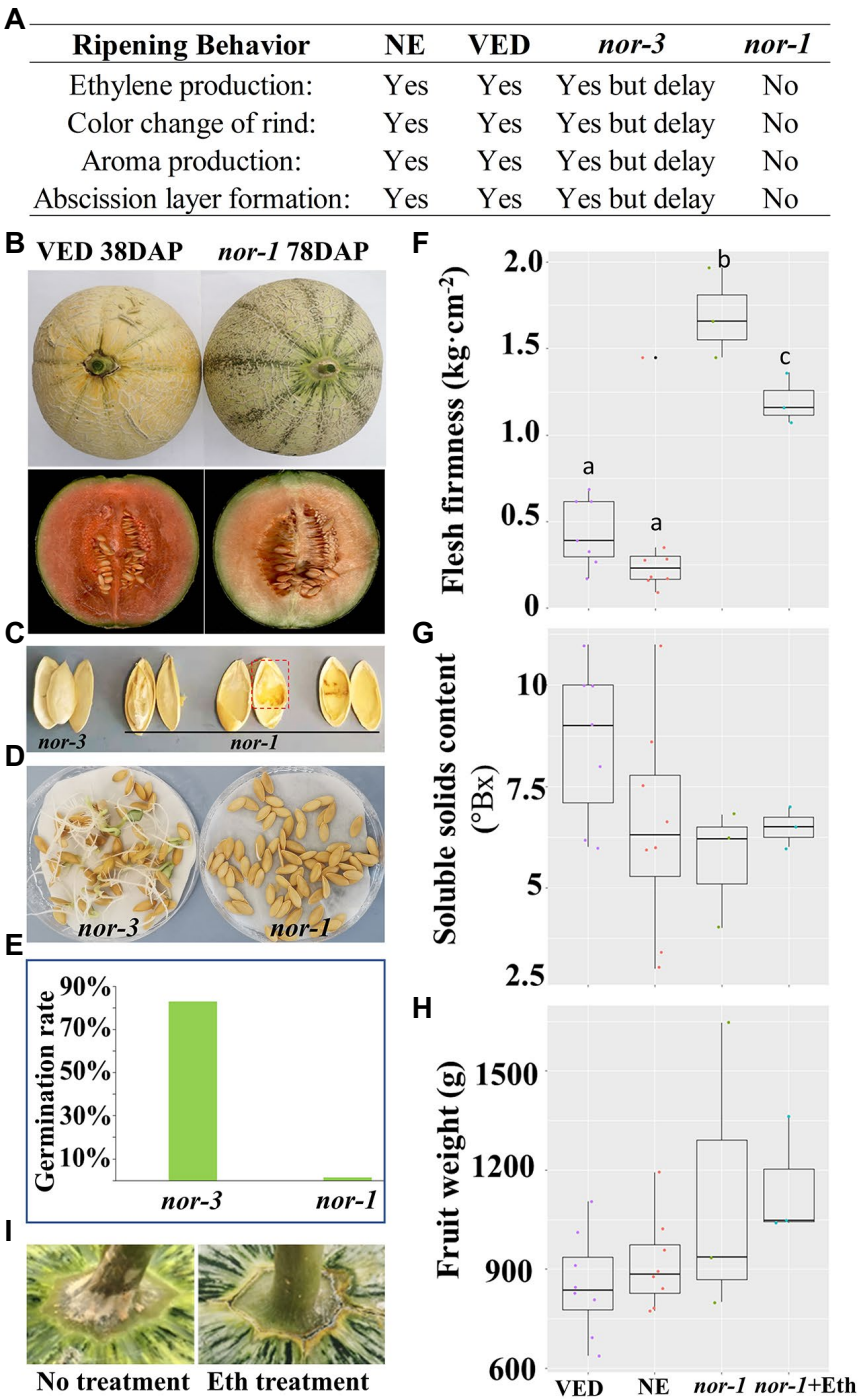
*CmNAC-NOR* knock out in *nor-1* blocks climacteric ripening. **(A)** Difference of ripening behavior among NE, VED, *nor-3,* and *nor-1*. **(B)** Fruit rind and flesh of VED and *nor-1* under natural ripening conditions. **(C)** Phenotypic comparison of seeds of *nor-3* and *nor-1* at 38 and 78 DAP, respectively. **(D,E)** Germination efficiency of *nor-3* and *nor-1* seeds. **(F-H)** Phenotypic comparison according to flesh firmness **(F)**, soluble solids content **(G)**, and fruit weight **(H)** between NE, VED, and *nor-1*. *nor-1* + Eth: *nor-1* fruit after ethylene treatment. **(I)** Abscission layer of *nor-1* appears after ethylene treatment. Lower case letters indicate significant differences (*p* < 0.05, *n* > 5).

The behavior of the *nor-1* mutant at the fruit ripening stage differed from that of the *nor*-3 mutant, with the progress of climacteric ripening totally blocked in *nor-1* in contrast to only being delayed in *nor-3.* Ethylene production, rind color change, aroma production, and abscission layer formation did not occur in *nor-1* (Figure 4A). As shown in Figure 4B, the external color of VED changed to yellow at 38 DAP, while the rind color of *nor-1* remained green at 78 DAP. Comparing flesh color and carotenoid content, *nor-1* flesh was slightly less orange than VED (Figure 4B); however, the total carotenoid content was not significantly different (data not shown). Surprisingly, *nor-1* seeds were not well developed (Figure 4C), resulting in an extremely low germination rate (1.25%; Figures 4D,E), while seed development was not affected in *nor-3* (Figure 4C), which had an 82.86% germination rate (Figures 4D,E). In addition, the flesh of *nor-1* was firmer than that of VED and NE fruits (Figure 4F). We found no significant difference in SSC (Figure 4G) or fruit weight (Figure 4H) between VED, NE, and *nor-1* fruits.

### Ethylene Treatment Did Not Recover Climacteric Ripening in *nor-1*

Given that ethylene plays a major role in the ripening of climacteric fruit (Ayub et al., 1996), we explored whether external ethylene treatment could induce ripening in *nor-1* mutants. The results showed that climacteric ripening was not induced after 1 week of external ethylene treatment. Rind color did not change to yellow (Figure 4B), fruits did not produce aroma, and flesh firmness slightly decreased but was still significantly more than VED and NE fruits (Figure 4F). We did not see a significant change in SSC (Figure 4G) or fruit weight (Figure 4H), but the abscission layer was induced 3 days after ethylene treatment (Figure 4I).

## Discussion

NAC transcription factors are a large gene family involved in plant development and the environment stress response (Guo and Gan, 2006; Nuruzzaman et al., 2013; Hernández and Sanan-Mishra, 2017). Among them, the *SlNAC-NOR* gene from tomato is well known as a key regulator in fruit ripening (Giovannoni, 2007). In previous studies, we have characterized melon *CmNAC-NOR*, a close homologue of *SlNAC-NOR* according to a phylogenetic analysis with NAC genes from several species, and demonstrated its involvement in fruit ripening with the delayed ripening phenotype of two TILLING mutant lines (Ríos et al., 2017). RNA-seq expression analysis of *CmNAC-NOR* in Ved (climacteric) and PS (non-climacteric) at different ripening stages showed that both lines have a similar expression profile during ripening (Supplementary Figure S2). In this study, we obtained two diploid *CmNAC-NOR*-disrupted melon plants with different edited sites (*nor-1* and *nor-3*) by using the CRISPR/Cas9 system (Figure 1). *nor-1* is a complete knock-out mutant where climacteric ripening behavior is almost absent, without ethylene or aroma production, a volatile profile similar to unripe fruit, absence of abscission layer, with no external color change (Figures 2, 4) and with low expression of several ripening-related genes (Supplementary Figure S1), suggesting that *CmNAC-NOR* plays a significant role in regulating fruit ripening in melon. *Nor-3* is a knock-down mutant with one amino acid deletion at the NAC (NAM, ATAF1,2, CUC2) domain, which causes around 8 days delay in ripening, but without affecting either fruit quality (Figure 3), nor gene expression of several ripening-related genes at harvest (Supplementary Figure S1), suggesting a potential way to control fruit ripening in melon by disrupting *CmNAC-NOR*. Moreover, in the heterozygous *nor-1,* there was also a 20 day delay in ethylene production (Figure 2A), suggesting that *nor-1* might have potential for extending the shelf life of fruit in melon breeding programs.

In tomato, fruit ripening is affected in both natural and CRISPR/Cas9 knock out mutants of *SlNAC-NOR* (Gao et al., 2020). However, the phenotype of fruit of *SlNAC-NOR* mutations (*CR-NOR*) induced by CRISPR/Cas9 has been found to be much less severe than the natural mutant *slnac-nor* (Wang et al., 2019; Gao et al., 2020). Mature fruit of the *slnac-nor* mutant does not produce an ethylene burst (Giovannoni, 2007; Adaskaveg et al., 2021) and has little carotenoid content (Giovannoni et al., 1995; Kumar et al., 2018), but *CR-NOR* fruits can produce ethylene and synthesize much more carotenoids than *slnac-nor* at the ripe stage (Gao et al., 2020). Therefore, *slnac-nor* was reported as a gain-of-function mutation (Gao et al., 2020) suggesting that *SlNAC-NOR* does not act as a master regulator but as a major gene controlling the ripening process (Wang et al., 2020a). In the VED climacteric melon, fruit ripening occurred around 35 ~ 40 DAP (Figure 2A) and was associated with a transient increase in autocatalytic ethylene production, accompanied by changes in rind and flesh color, flesh firmness, sugar content, and aroma production (Pereira et al., 2018, 2020; Mayobre et al., 2021). In the *nor-3* knock down mutant, the mutation was located within the DNA/protein binding region of the NAC-NOR transcription factor, so we could expect a reduction of its binding affinity, affecting its regulation of ripening-related genes. As it was expected, we observed a delay in ripening (ethylene and aroma production), suggesting that the mutated protein was still functional but less efficient (Figure 2). This phenotype agreed with Ríos et al. (2017), where non-synonymous mutations in the conserved NAC domain region caused a delay in fruit ripening. Concerning fruit quality, we demonstrated that even though the fruit *nor-3* ripened later, it was able to produce a peak of ethylene similar to VED, which probably allowed the *nor-3* fruit attain the same quality parameters as VED such as VOCs profile or flesh firmness (Figure 3), that are ethylene-dependent or partially dependent (Pech et al., 2008). This was confirmed by the transcription analysis of genes involved in ethylene biosynthesis, ester production, and fruit softening, where *nor-3* showed similar expression data as VED at harvest. In the complete knock-out mutant *nor-1*, ethylene production was blocked (Figure 2A), the expression of *CmACS1 and CmACO1* was repressed (Supplementary Figure S1), the aroma component changed (Figure 2C), *CmAAT1* and *CmADH2* were downregulated (Supplementary Figure S1), the rind color did not change from green to yellow (Figure 4B), no abscission layer formed, the flesh was firmer than VED (Figure 4F), and two *CmPGs* were downregulated (Supplementary Figure S1). The flesh color of *nor-1* seemed visually less orange than VED (Figure 4B), although we did not detect significant differences in carotenoid content, nor in *CmOr* expression (Supplementary Figure S1). It remains to be tested if the composition of individual carotenoid compounds is altered in the mutant, without modifying the total carotenoid content. Our findings suggest that *nor-1* resembles the tomato phenotypes of *slnac-nor* and *CR-NOR* mutants. Unlike the tomato CRISPR mutant *CR-NOR*, the main climacteric ripening components were almost blocked in *nor-1* melon. In addition, *nor-1* was insensitive to external ethylene treatment (Figure 4), except for abscission layer formation, suggesting that *CmNAC-NOR* gene is a major key regulator of fruit ripening in melon.

The different phenotypes of tomato and melon CRISPR *NAC-NOR* mutants might be explained by their different editing patterns. Although they lose the transcriptional regulation region, their NAC domain is altered at different levels. The NAC domain contains five subdomains (A-E) that play an important role in DNA-binding (Kikuchi et al., 2000; Ernst et al., 2004). The *slnac-nor* mutant has been found to contain a complete NAC domain, resulting in a gain-of-function mutation, while the *CR-NOR* mutant produced a truncated protein of 47 aa, which lost NAC subdomains B-E, but still had the NAC subdomain A (Gao et al., 2020). Here, the editing of *nor-1* started from the NAC subdomain A (Figure 1E), so the whole NAC domain was affected in *nor-1,* resulting in a loss-of-function mutation, whereas *nor-3* lost a single amino acid at NAC subdomain A, resulting in a delay of ripening.

Fruit flavor is an important trait as it affects consumer preferences. Volatile esters are major contributors to fruit flavor giving the fruity aroma to climacteric melons (El Hadi et al., 2013). Compared to the controls, the esters content was dramatically reduced in *nor-1* and the content of aldehydes was increased, which explain the green, fresh aroma of these fruits. The *nor-1* VOCs profile was more similar to unripe melons or to non-climacteric melons such as *inodorus* types (Mayobre et al., 2021) than to the VED profile. These results suggest that *CmNAC-NOR* could be involved in the regulation of the *AAT* genes, which are known to be ethylene-dependent and are responsible for volatile ester formation (El-Sharkawy et al., 2005; Cao et al., 2021). As expected, when we measured the expression of *CmAAT1,* we observed that it was downregulated in the *nor-1* mutant, compared to the control lines and the *nor-3* mutant. This is also consistent with a recent study that reported that the NAC transcription factor *PpNAC1* (with homology to *SlNAC-NOR*) regulates fruit flavor ester biosynthesis in peach by activating *PpAAT1* expression (Cao et al., 2021). However, to demonstrate that *CmNAC-NOR* directly binds *CmAAT1*, further experiments are needed.

An unexpected phenotype of *nor-1* was that seeds were not well developed (Figure 4C). This phenotype has not previously been reported in the tomato *NOR* mutant or in other NAC genes in species such as peach (Pirona et al., 2013), apple (Yeats et al., 2019), and strawberry (Martín-Pizarro et al., 2021). However, there are some reports suggesting that NAC transcription factors regulate seed development and play a role in seed germination (Kim et al., 2008; Park et al., 2011; Wang et al., 2021; Liu et al., 2022). In a recent study, knock out of the *ClNAC68* gene in watermelon delayed seed maturation and germination, but the germination rate was not affected (Wang et al., 2021), suggesting that there are additional NAC genes with diverse functions that regulate seed development. In tomato, *NOR-like1* has been shown to be a positive regulator of fruit ripening; CRISPR/Cas9 mutants of *NOR-like1* delayed fruit ripening and seriously affected seed development, reducing the number and weight of seeds, which showed poor germination (Gao et al., 2018). However, the target genes associated with seed development are still unknown. Only a recent study in grape showed that the NAC domain gene *VvNAC26*, which positively regulates ethylene and ABA-related genes to influence seed and fruit development, interacts with the transcription factor *VvMADS9* (Zhang et al., 2021).

Non-climacteric melon cultivars as Piel de Sapo (PS), a variety belonging to the *inodorous* group in the *melo* subspecies, produce low amount of ethylene, insufficient to trigger the climacteric response and do not abscise when ripe. Climacteric varieties as VED, a variety from the *cantaloupensis* group in the *melo* subspecies, show a typical climacteric fruit ripening behavior, with a sharp ethylene peak and noticeable related climacteric traits as abscission layer formation at around 35 DAP. There are no significant differences between both types in soluble solid content nor firmness of the flesh (Pereira et al., 2020), but the fruit volatiles produced by both types are different (Mayobre et al., 2021). Three climacteric QTLs involved in fruit ripening have been characterized in melon, *ETHQB3.5*, *ETHQV6.3,* and *ETHQV8.1* (Vegas et al., 2013; Pereira et al., 2020). So far, only the causal gene for *ETHQV6.3* has been identified (*CmNAC-NOR*, Ríos et al., 2017). Introgression lines carrying the climacteric allele of each of the three QTL in the non-climacteric PS background are able to induce a very mild climacteric response (Vegas et al., 2013; Pereira et al., 2020). However, when combined in pairs or the three of them together, they interact epistatically, producing a dramatic climacteric effect in the non-climacteric background (unpublished). These data suggest that the non-climacteric PS may be impaired in ethylene production due to variations in more than one gene, and that the combination of two or more genes is necessary to rescue the typical climacteric response. In addition, the non-climacteric allele of *ETHQV8.1* in the VED background delays ripening but does not result in a strong non-climacteric phenotype (Pereira et al., 2020). A plausible hypothesis is that at least these three genes/QTL are responsible of the ripening differences between non-climacteric melons from the *inodorus* group and the climacteric cantaloupe type. However, the complex molecular mechanisms cannot be yet understood until the causal genes of the other two QTL are identified. Still, we cannot rule out that additional genes are responsible for conferring a non-climacteric response in other non-climacteric melon types phylogenetically distant from PS.

In this study, we provide evidence that supports *CmNAC-NOR* as a key player in regulating climacteric fruit ripening in melon. As a master regulator, *CmNAC-NOR* independently mediates many ripening-associated traits. Our findings also suggest that *CmNAC-NOR* can be a potential target in breeding programs to modulate fruit maturation and shelf life in melon.

## Data Availability Statement

The original contributions presented in the study are included in the article/Supplementary Material, further inquiries can be directed to the corresponding authors.

## Author Contributions

BL did the experimental work and data analysis and wrote the original draft of the manuscript. MS grew the plants, phenotyped the ripening behavior, and measured the ethylene content. CM performed the expression and volatile experiments. AM-H supervised the CRISPR-Cas9 and genetic transformation experiments. MP and JG-M designed and supervised the work and reviewed and edited the manuscript. All authors contributed to the article and approved the submitted version.

## Funding

This work was supported by grants AGL2015–64625-C2–1-R and RTI2018-097665-B-C2 funded by MCIN/AEI/10.13039/501100011033 and by “ERDF A way of making Europe,” the Severo Ochoa Programme for Centres of Excellence in R&D 2016–2010 (SEV-2015-0533) funded by MCIN/AEI/10.13039/501100011033, the CERCA Programme/Generalitat de Catalunya and 2017 SGR 1319 grant from the Generalitat de Catalunya to JG-M. BL was also supported by grants from Youth Project of National Natural Science Foundation of China (31902035), The International Postdoctoral Exchange Fellowship Program of China (20170053), and a postdoctoral grant from the Severo Ochoa Programme for Centres of Excellence in R&D 2016–2010 (SEV-2015-0533). MS was supported by a grant BES-2017-079956 funded by MCIN/AEI/ 10.13039/501100011033 and by “ESF Investing in your future”. CM was supported by FI grant from the Secretaria d’Universitats i Recerca del Departament d’Empresa i Coneixement de la Generalitat de Catalunya and the co-funding of the European Social Fund (ESF)—“ESF is investing in your future.”

## Conflict of Interest

The authors declare that they have no known competing financial interests or personal relationships that could have appeared to influence the research reported in this paper.

## Publisher’s Note

All claims expressed in this article are solely those of the authors and do not necessarily represent those of their affiliated organizations, or those of the publisher, the editors and the reviewers. Any product that may be evaluated in this article, or claim that may be made by its manufacturer, is not guaranteed or endorsed by the publisher.

## References

[ref1] Adaskaveg J. A. Silva C. J. Huang P. Blanco-Ulate B. (2021). Single and double mutations in tomato ripening transcription factors have distinct effects on fruit development and quality traits. Front. Plant Sci. 12:647035. doi: 10.3389/fpls.2021.647035, PMID: 33986762 PMC8110730

[ref2] Alexander L. Grierson D. (2002). Ethylene biosynthesis and action in tomato: a model for climacteric fruit ripening. J. Exp. Bot. 53, 2039–2055. doi: 10.1093/jxb/erf072, PMID: 12324528

[ref3] Ayub R. Guis M. Amor M. B. Gillot L. Roustan J. P. Latché A.. (1996). Expression of *ACC* oxidase antisense gene inhibits ripening of cantaloupe melon fruits. Nat. Biotechnol. 14, 862–866. doi: 10.1038/nbt0796-862, PMID: 9631011

[ref4] Bemer M. Karlova R. Ballester A. R. Tikunov Y. M. Bovy A. G. Wolters-Arts M.. (2012). The tomato FRUITFULL homologs TDR4/FUL1 and MBP7/FUL2 regulate ethylene-independent aspects of fruit ripening. Plant Cell 24, 4437–4451. doi: 10.1105/tpc.112.103283, PMID: 23136376 PMC3531844

[ref5] Blankenship S. M. Dole J. M. (2003). 1-Methylcyclopropene: a review. Postharvest Biol. Technol. 28, 1–25. doi: 10.1016/S0925-5214(02)00246-6

[ref6] Bouzayen M. Latché A. Nath P. Pech J. C. (2010). “Mechanism of fruit ripening,” in Plant Developmental Biology-Biotechnological Perspectives. eds. Pua E. C. Davey M. R. (New York: Springer), 319–339.

[ref7] Cao X. Wei C. Duan W. Gao Y. Kuang J. Liu M.. (2021). Transcriptional and epigenetic analysis reveals that NAC transcription factors regulate fruit flavor ester biosynthesis. Plant J. 106, 785–800. doi: 10.1111/tpj.15200, PMID: 33595854

[ref8] Castelblanque L. Marfa V. Claveria E. Martinez I. Perez-Grau L. Dolcet-Sanjuan R. (2008). “Improving the genetic transformation efficiency of *Cucumis melo* subsp. Melo ‘Piel de Sapo’via Agrobacterium. Avignon (France).” in Cucurbitaceae 2008 May 21-24, Proceedings of the IXth EUCARPIA Meeting on Genetics and Breeding of Cucurbitaceae. ed. Pitrat M., 627–632.

[ref9] Chai Y. Jia H. Li C. Dong Q. Shen Y. (2011). FaPYR1 is involved in strawberry fruit ripening. J. Exp. Bot. 62, 5079–5089. doi: 10.1093/jxb/err207, PMID: 21778181

[ref10] Chen J. Liu D. Jiang Y. Zhao M. Shan W. Kuang J.. (2011). Molecular characterization of a strawberry *FaASR* gene in relation to fruit ripening. PLoS One 6:e24649. doi: 10.1371/journal.pone.0024649, PMID: 21915355 PMC3167850

[ref11] Choi Y. Chan A. P. (2015). PROVEAN web server: a tool to predict the functional effect of amino acid substitutions and indels. Bioinformatics 31, 2745–2747. doi: 10.1093/bioinformatics/btv195, PMID: 25851949 PMC4528627

[ref12] Chung M.-Y. Vrebalov J. Alba R. Lee J. McQuinn R. Chung J.-D.. (2010). A tomato (*Solanum lycopersicum*) APETALA2/ERF gene, SlAP2a, is a negative regulator of fruit ripening. Plant J. 64, 936–947. doi: 10.1111/j.1365-313X.2010.04384.x 21143675

[ref13] Civello P. M. Powell A. L. Sabehat A. Bennett A. B. (1999). An expansin gene expressed in ripening strawberry fruit. Plant Physiol. 121, 1273–1279. doi: 10.1104/pp.121.4.1273, PMID: 10594114 PMC59494

[ref14] Eduardo I. Arús P. Monforte A. J. (2005). Development of a genomic library of near isogenic lines (NILs) in melon (*Cucumis melo* L.) from the exotic accession PI161375. Theor. Appl. Genet. 112, 139–148. doi: 10.1007/s00122-005-0116-y, PMID: 16208502

[ref15] El Hadi M. A. M. Zhang F. J. Wu F. F. Zhou C. H. Tao J. (2013). Advances in fruit aroma volatile research. Molecules 18, 8200–8229. doi: 10.3390/molecules18078200, PMID: 23852166 PMC6270112

[ref16] El-Sharkawy I. Manríquez D. Flores F. B. Regad F. Bouzayen M. Latche A.. (2005). Functional characterization of a melon alcohol acyl-transferase gene family involved in the biosynthesis of ester volatiles. Identification of the crucial role of a threonine residue for enzyme activity. Plant Mol. Biol. 59, 345–362. doi: 10.1007/s11103-005-8884-y, PMID: 16247561

[ref17] Ernst H. A. Nina Olsen A. Skriver K. Larsen S. Lo Leggio L. (2004). Structure of the conserved domain of ANAC, a member of the NAC family of transcription factors. EMBO Rep. 5, 297–303. doi: 10.1038/sj.embor.7400093, PMID: 15083810 PMC1299004

[ref18] Ezura H. Amagai H. Yoshioka K. Oosawa K. (1992). Highly frequent appearance of tetraploidy in regenerated plants, a universal phenomenon, in tissue cultures of melon (*Cucumis melo* L.). Plant Sci. 85, 209–213. doi: 10.1016/0168-9452(92)90117-5

[ref19] Ezura H. Owino W. O. (2008). Melon, an alternative model plant for elucidating fruit ripening. Plant Sci. 175, 121–129. doi: 10.1016/j.plantsci.2008.02.004

[ref20] Flores F. Ben Amor M. Jones B. Pech J. Bouzayen M. Latché A.. (2001). The use of ethylene-suppressed lines to assess differential sensitivity to ethylene of the various ripening pathways in cantaloupe melons. Physiol. Plant. 113, 128–133. doi: 10.1034/j.1399-3054.2001.1130117.x

[ref21] Gao Y. Wei W. Fan Z. Zhao X. Zhang Y. Jing Y.. (2020). Re-evaluation of the nor mutation and the role of the NAC-NOR transcription factor in tomato fruit ripening. J. Exp. Bot. 71, 3560–3574. doi: 10.1093/jxb/eraa131, PMID: 32338291 PMC7307841

[ref22] Gao Y. Wei W. Zhao X. Tan X. Fan Z. Zhang Y.. (2018). A NAC transcription factor, NOR-like1, is a new positive regulator of tomato fruit ripening. Hort. Res. 5:75. doi: 10.1038/s41438-018-0111-5, PMID: 30588320 PMC6303401

[ref23] Gao Y. Zhu N. Zhu X. Wu M. Jiang C.-Z. Grierson D.. (2019). Diversity and redundancy of the ripening regulatory networks revealed by the fruitENCODE and the new CRISPR/Cas9 CNR and NOR mutants. Hort. Res. 6:39. doi: 10.1038/s41438-019-0122-x, PMID: 30774962 PMC6370854

[ref305] Gapper N. E. McQuinn R. P. Giovannoni J. J. (2013). Molecular and genetic regulation of fruit ripening. Plant Mol. Biol. 82, 575–591. doi: 10.1007/s11103-013-0050-3 23585213

[ref24] García-Almodóvar R. Gosalvez B. Aranda M. A. Burgos L. (2017). Production of transgenic diploid *Cucumis melo* plants. Plant Cell Tissue Organ Cult. 130, 323–333. doi: 10.1007/s11240-017-1227-2

[ref25] Giovannoni J. J. (2007). Fruit ripening mutants yield insights into ripening control. Curr. Opin. Plant Biol. 10, 283–289. doi: 10.1016/j.pbi.2007.04.008, PMID: 17442612

[ref26] Giovannoni J. J. Noensie E. N. Ruezinsky D. M. Lu X. Tracy S. L. Ganal M. W.. (1995). Molecular genetic analysis of the ripening-inhibitor and non-ripening loci of tomato: a first step in genetic map-based cloning of fruit ripening genes. Mol. Gen. Genet. 248, 195–206. doi: 10.1007/BF02190801, PMID: 7651343

[ref27] Guo Y. Gan S. (2006). AtNAP, a NAC family transcription factor, has an important role in leaf senescence. Plant J. 46, 601–612. doi: 10.1111/j.1365-313X.2006.02723.x, PMID: 16640597

[ref28] Hadfield K. A. Rose J. C. K. Yaver D. S. Berka R. M. Bennett A. B. (1998). Polygalacturonase gene expression in ripe melon fruit supports a role for polygalacturonase in ripening-associated pectin disassembly. Plant Physiol. 117, 363–373. doi: 10.1104/pp.117.2.363, PMID: 9625689 PMC34956

[ref29] Hamilton A. Lycett G. Grierson D. (1990). Antisense gene that inhibits synthesis of the hormone ethylene in transgenic plants. Nature 346, 284–287. doi: 10.1038/346284a0

[ref30] Hernández Y. Sanan-Mishra N. (2017). miRNA mediated regulation of NAC transcription factors in plant development and environment stress response. Plant Gene 11, 190–198. doi: 10.1016/j.plgene.2017.05.013

[ref31] Hiwasa-Tanase K. Ezura H. (2014). “Climacteric and non-climacteric ripening,” in Fruit Ripening, Physiology, Signalling and Genomics. eds. Nath P. Bouzayen M. Mattoo A. K. Pech J. C. (United Kingdom: CABI), 1–14.

[ref32] Itkin M. Seybold H. Breitel D. Rogachev I. Meir S. Aharoni A. (2009). TOMATO AGAMOUS-LIKE 1 is a component of the fruit ripening regulatory network. Plant J. 60, 1081–1095. doi: 10.1111/j.1365-313X.2009.04064.x, PMID: 19891701

[ref33] Ito Y. Nishizawa-Yokoi A. Endo M. Mikami M. Shima Y. Nakamura N.. (2017). Re-evaluation of the rin mutation and the role of RIN in the induction of tomato ripening. Nat. Plants 3, 866–874. doi: 10.1038/s41477-017-0041-5, PMID: 29085071

[ref34] Jia H. Lu D. Sun J. Li C. Xing Y. Qin L.. (2013). Type 2C protein phosphatase ABI1 is a negative regulator of strawberry fruit ripening. J. Exp. Bot. 64, 1677–1687. doi: 10.1093/jxb/ert028, PMID: 23404898 PMC3617833

[ref35] Karlova R. Rosin F. M. Busscher-Lange J. Parapunova V. Do P. T. Fernie A. R.. (2011). Transcriptome and metabolite profiling show that APETALA2a is a major regulator of tomato fruit ripening. Plant Cell 23, 923–941. doi: 10.1105/tpc.110.081273, PMID: 21398570 PMC3082273

[ref36] Katz E. Lagunes P. M. Riov J. Weiss D. Goldschmidt E. E. (2004). Molecular and physiological evidence suggests the existence of a system II-like pathway of ethylene production in non-climacteric Citrus fruit. Planta 219, 243–252. doi: 10.1007/s00425-004-1228-3, PMID: 15014996

[ref37] Kikuchi K. Ueguchi-Tanaka M. Yoshida K. Nagato Y. Matsusoka M. Hirano H. Y. (2000). Molecular analysis of the *NAC* gene family in rice. Mol. Gen. Genet. MGG 262, 1047–1051. doi: 10.1007/PL00008647 10660065

[ref38] Kim S. G. Lee A. K. Yoon H. K. Park C. M. (2008). A membrane-bound NAC transcription factor NTL8 regulates gibberellic acid-mediated salt signaling in Arabidopsis seed germination. Plant J. 55, 77–88. doi: 10.1111/j.1365-313X.2008.03493.x, PMID: 18363782

[ref39] Kumar R. Tamboli V. Sharma R. Sreelakshmi Y. (2018). NAC-NOR mutations in tomato Penjar accessions attenuate multiple metabolic processes and prolong the fruit shelf life. Food Chem. 259, 234–244. doi: 10.1016/j.foodchem.2018.03.135, PMID: 29680049

[ref40] Liu G.-S. Li H.-L. Grierson D. Fu D.-Q. (2022). NAC transcription factor family regulation of fruit ripening and quality: a review. Cell 11:525. doi: 10.3390/cells11030525 PMC8834055 35159333

[ref41] Livak K. J. Schmittgen T. D. (2001). Analysis of relative gene expression data using real-time quantitative PCR and the 2(-Delta Delta C(T)) method. Methods 25, 402–408. doi: 10.1006/meth.2001.1262 11846609

[ref42] Lü P. Yu S. Zhu N. Chen Y.-R. Zhou B. Pan Y.. (2018). Genome encode analyses reveal the basis of convergent evolution of fleshy fruit ripening. Nat. Plants 4, 784–791. doi: 10.1038/s41477-018-0249-z, PMID: 30250279

[ref43] Manning K. Tör M. Poole M. Hong Y. Thompson A. J. King G. J.. (2006). A naturally occurring epigenetic mutation in a gene encoding an SBP-box transcription factor inhibits tomato fruit ripening. Nat. Genet. 38, 948–952. doi: 10.1038/ng1841, PMID: 16832354

[ref44] Manríquez D. El-Sharkawy I. Flores F. El-Yahyaoui F. Regad F. Bouzayen M.. (2006). Two highly divergent alcohol dehydrogenases of melon exhibit fruit ripening-specific expression and distinct biochemical characteristics. Plant Mol. Biol. 61, 675–685. doi: 10.1007/s11103-006-0040-9, PMID: 16897483

[ref45] Martín-Pizarro C. Vallarino J. G. Osorio S. Meco V. Urrutia M. Pillet J.. (2021). The NAC transcription factor FaRIF controls fruit ripening in strawberry. Plant Cell 33, 1574–1593. doi: 10.1093/plcell/koab070, PMID: 33624824 PMC8254488

[ref46] Mayobre C. Pereira L. Eltahiri A. Bar E. Lewinsohn E. Garcia-Mas J.. (2021). Genetic dissection of aroma biosynthesis in melon and its relationship with climacteric ripening. Food Chem. 353:129484. doi: 10.1016/j.foodchem.2021.129484 33812162

[ref47] McMurchie E. McGlasson W. Eaks I. (1972). Treatment of fruit with propylene gives information about the biogenesis of ethylene. Nature 237, 235–236. doi: 10.1038/237235a0, PMID: 4557321

[ref48] Moreno E. Obando J. M. Dos-Santos N. Fernández-Trujillo J. P. Monforte A. J. Garcia-Mas J. (2008). Candidate genes and QTLs for fruit ripening and softening in melon. Theor. Appl. Genet. 116, 589–602. doi: 10.1007/s00122-007-0694-y, PMID: 18094954

[ref49] Nishiyama K. Guis M. Rose J. K. Kubo Y. Bennett K. A. Wangjin L.. (2007). Ethylene regulation of fruit softening and cell wall disassembly in Charentais melon. J. Exp. Bot. 58, 1281–1290. doi: 10.1093/jxb/erl283, PMID: 17308329

[ref50] Nuruzzaman M. Sharoni A. M. Kikuchi S. (2013). Roles of NAC transcription factors in the regulation of biotic and abiotic stress responses in plants. Front. Microbiol. 4:248. doi: 10.3389/fmicb.2013.00248 24058359 PMC3759801

[ref51] Obando-Ulloa J. M. Moreno E. Garcia-Mas J. Nicolai B. Lammertyn J. Monforte A. J.. (2008). Climacteric or non-climacteric behavior in melon fruit: 1. Aroma volatiles. Postharvest Biol. Technol. 49, 27–37. doi: 10.1016/j.postharvbio.2007.11.004

[ref52] Oliveros J. C. Franch M. Tabas-Madrid D. San-León D. Montoliu L. Cubas P.. (2016). Breaking-Cas—interactive design of guide RNAs for CRISPR-Cas experiments for ENSEMBL genomes. Nucleic Acids Res. 44, W267–W271. doi: 10.1093/nar/gkw407, PMID: 27166368 PMC4987939

[ref53] Osorio S. Scossa F. Fernie A. (2013). Molecular regulation of fruit ripening. Front. Plant Sci. 4:198. doi: 10.3389/fpls.2013.00198 23785378 PMC3682129

[ref54] Park J. Kim Y. S. Kim S. G. Jung J. H. Woo J. C. Park C. M. (2011). Integration of auxin and salt signals by the NAC transcription factor NTM2 during seed germination in Arabidopsis. Plant Physiol. 156, 537–549. doi: 10.1104/pp.111.177071, PMID: 21450938 PMC3177257

[ref55] Paul V. Pandey R. Srivastava G. C. (2012). The fading distinctions between classical patterns of ripening in climacteric and non-climacteric fruit and the ubiquity of ethylene—an overview. J. Food Sci. Technol. 49, 1–21. doi: 10.1007/s13197-011-0293-4, PMID: 23572821 PMC3550874

[ref56] Payasi A. Sanwal G. (2010). Ripening of climacteric fruits and their control. J. Food Biochem. 34, 679–710. doi: 10.1111/j.1745-4514.2009.00307.x

[ref57] Pech J.-C. Bouzayen M. Latché A. (2008). Climacteric fruit ripening: ethylene-dependent and independent regulation of ripening pathways in melon fruit. Plant Sci. 175, 114–120. doi: 10.1016/j.plantsci.2008.01.003

[ref58] Pereira L. Pujol M. Garcia-Mas J. Phillips M. A. (2017). Non-invasive quantification of ethylene in attached fruit headspace at 1 p.p.b. by gas chromatography-mass spectrometry. Plant J. 91, 172–183. doi: 10.1111/tpj.13545, PMID: 28370685

[ref59] Pereira L. Ruggieri V. Pérez S. Alexiou K. G. Fernández M. Jahrmann T.. (2018). QTL mapping of melon fruit quality traits using a high-density GBS-based genetic map. BMC Plant Biol. 18:324. doi: 10.1186/s12870-018-1537-5, PMID: 30509167 PMC6278158

[ref60] Pereira L. Santo Domingo M. Ruggieri V. Argyris J. Phillips M. A. Zhao G.. (2020). Genetic dissection of climacteric fruit ripening in a melon population segregating for ripening behavior. Hort. Res. 7, 1–18. doi: 10.1038/s41438-020-00411-z PMC7603510 33328460

[ref61] Périn C. Gomez-Jimenez M. Hagen L. Dogimont C. Pech J. C. Latché A.. (2002). Molecular and genetic characterization of a non-climacteric phenotype in melon reveals two loci conferring altered ethylene response in fruit. Plant Physiol. 129, 300–309. doi: 10.1104/pp.010613, PMID: 12011360 PMC155893

[ref62] Pirona R. Eduardo I. Pacheco I. Linge C. D. S. Miculan M. Verde I.. (2013). Fine mapping and identification of a candidate gene for a major locus controlling maturity date in peach. BMC Plant Biol. 13, 1–13. doi: 10.1186/1471-2229-13-166 24148786 PMC3854093

[ref63] R Core Team (2020). R: A Language and Environment for Statistical Computing. R Foundation for Statistical Computing, Vienna, Austria.

[ref64] Ríos P. Argyris J. Vegas J. Leida C. Kenigswald M. Tzuri G.. (2017). ETHQV6.3 is involved in melon climacteric fruit ripening and is encoded by a NAC domain transcription factor. Plant J. 91, 671–683. doi: 10.1111/tpj.13596, PMID: 28493311

[ref65] Saladié M. Canizares J. Phillips M. A. Rodriguez-Concepcion M. Larrigaudiere C. Gibon Y.. (2015). Comparative transcriptional profiling analysis of developing melon (*Cucumis melo* L.) fruit from climacteric and non-climacteric varieties. BMC Genomics 16:440. doi: 10.1186/s12864-015-1649-3 26054931 PMC4460886

[ref200] Vegas J. Garcia-Mas J. Monforte A. J. (2013). Interaction between QTLs induces an advance in ethylene biosynthesis during melon fruit ripening. Theor. Appl. Genet. 126, 1531–1554. doi: 10.1007/s00122-013-2071-3, PMID: 23443139

[ref66] Vrebalov J. Pan I. L. Arroyo A. J. M. McQuinn R. Chung M. Poole M.. (2009). Fleshy fruit expansion and ripening are regulated by the tomato SHATTERPROOF gene TAGL1. Plant Cell 21, 3041–3062. doi: 10.1105/tpc.109.066936, PMID: 19880793 PMC2782289

[ref67] Vrebalov J. Ruezinsky D. Padmanabhan V. White R. Medrano D. Drake R.. (2002). A MADS-box gene necessary for fruit ripening at the tomato ripening-inhibitor (rin) locus. Science 296, 343–346. doi: 10.1126/science.1068181, PMID: 11951045

[ref68] Wang R. Angenent G. C. Seymour G. de Maagd R. A. (2020a). Revisiting the role of master regulators in tomato ripening. Trends Plant Sci. 25, 291–301. doi: 10.1016/j.tplants.2019.11.005, PMID: 31926765

[ref69] Wang R. da Rocha Tavano E. C. Lammers M. Martinelli A. P. Angenent G. C. de Maagd R. A. (2019). Re-evaluation of transcription factor function in tomato fruit development and ripening with CRISPR/Cas9-mutagenesis. Sci. Rep. 9, 1–10. doi: 10.1038/s41598-018-38170-6 30737425 PMC6368595

[ref70] Wang R. Lammers M. Tikunov Y. Bovy A. G. Angenent G. C. de Maagd R. A. (2020b). The *rin*, *nor* and *Cnr* spontaneous mutations inhibit tomato fruit ripening in additive and epistatic manners. Plant Sci. 294:110436. doi: 10.1016/j.plantsci.2020.110436, PMID: 32234221

[ref71] Wang J. Wang Y. Zhang J. Ren Y. Li M. Tian S.. (2021). The NAC transcription factor ClNAC68 positively regulates sugar content and seed development in watermelon by repressing ClINV and ClGH3. 6. Hort. Res. 8, 1–14. doi: 10.1038/s41438-021-00649-1 PMC8484586 34593776

[ref72] Watkins C. B. (2006). The use of 1-methylcyclopropene (1-MCP) on fruits and vegetables. Biotechnol. Adv. 24, 389–409. doi: 10.1016/j.biotechadv.2006.01.005 16530376

[ref73] Yeats T. H. Migicovsky Z. Watts S. Song J. Forney C. F. Burgher-MacLellan K.. (2019). Allelic diversity of NAC18. 1 is a major determinant of fruit firmness and harvest date in apple (*Malus domestica*). bioRxiv [Preprint].

[ref74] Zhang S. Dong R. Wang Y. Li X. Ji M. Wang X. (2021). NAC domain gene VvNAC26 interacts with VvMADS9 and influences seed and fruit development. Plant Physiol. Biochem. 164, 63–72. doi: 10.1016/j.plaphy.2021.04.031, PMID: 33965765

