# Supplementary figures and images for: Knock-Out of CmNAC-NOR Affects Melon Climacteric Fruit Ripening
Source: Front Plant Sci. 2022 Jun 10;13:878037. doi: 10.3389/fpls.2022.878037 (PMC9226586; doi:10.3389/fpls.2022.878037)

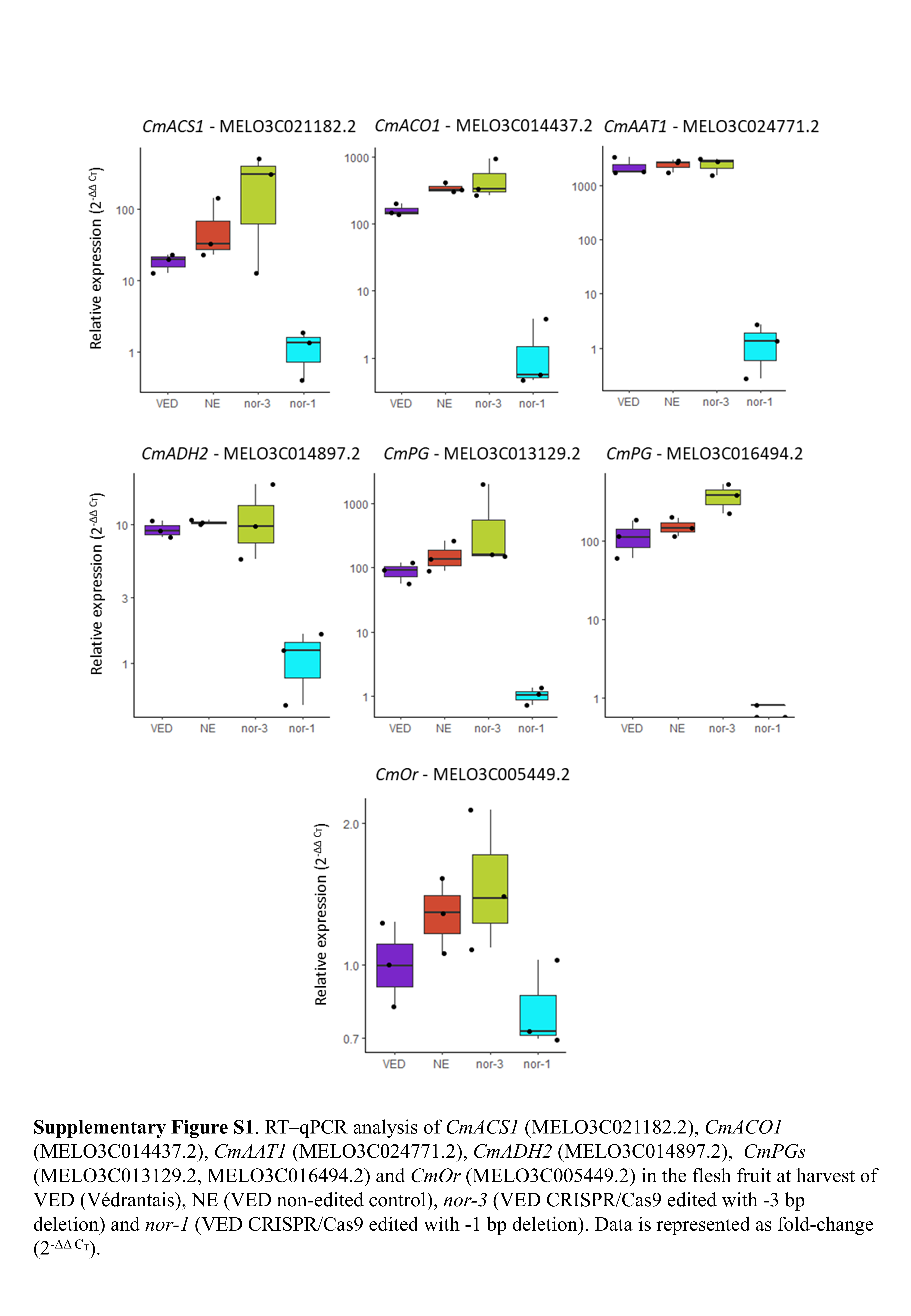

Supplement: Supplementary file 3 [file Image_1.TIF]

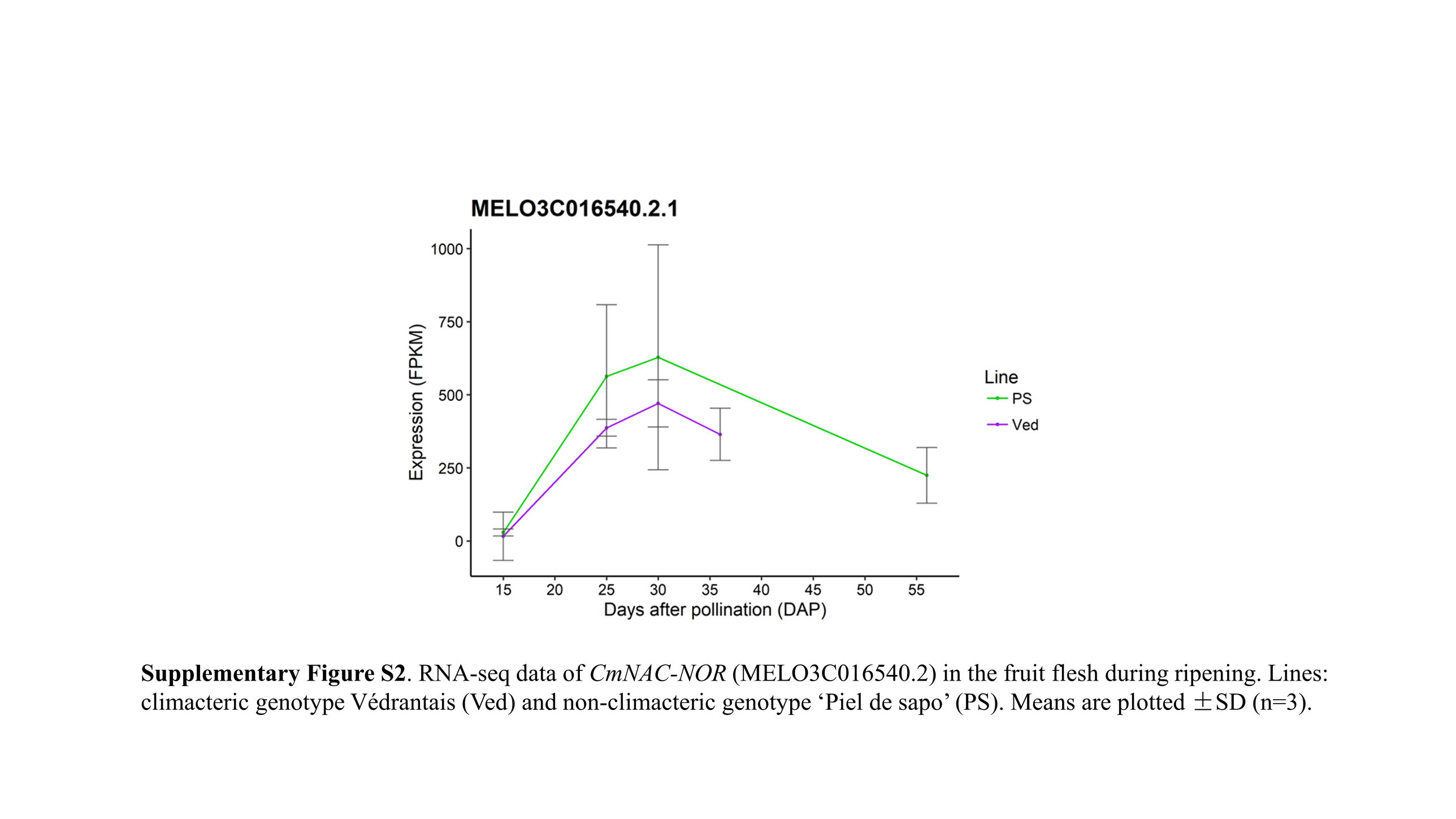

Supplement: Supplementary file 4 [file Image_2.TIF]
